# Breastfeeding Interpersonal Communication, Mobile Phone Support, and Mass Media Messaging Increase Exclusive Breastfeeding at 6 and 24 Weeks Among Clients of Private Health Facilities in Lagos, Nigeria

**DOI:** 10.1093/jn/nxab450

**Published:** 2022-01-07

**Authors:** Valerie L Flax, Abiodun Ipadeola, Courtney H Schnefke, Uche Ralph-Opara, Olatoun Adeola, Susan Edwards, Sujata Bose, Alice O Brower

**Affiliations:** RTI International, Research Triangle Park, NC, USA; Datametrics Associates Ltd., Abuja, Nigeria; RTI International, Research Triangle Park, NC, USA; Alive & Thrive Nigeria, FHI 360, Lagos, Nigeria; Equitable Health Access Initiative, Lagos, Nigeria; RTI International, Research Triangle Park, NC, USA; Alive & Thrive, FHI Solutions, Washington, DC, USA; RTI International, Research Triangle Park, NC, USA

**Keywords:** breastfeeding, health workers, mHealth, interpersonal communication, behavior change communication

## Abstract

**Background:**

Although most health facilities in urban Nigeria are privately owned, interventions to promote optimal breastfeeding practices in private facilities have not previously been implemented.

**Objectives:**

We tested the impact of a breastfeeding promotion intervention on early initiation of breastfeeding and exclusive breastfeeding among clients of private facilities in Lagos, Nigeria.

**Methods:**

The intervention included training for health-care providers on the Baby-Friendly Hospital Initiative and breastfeeding counseling skills, provision of interpersonal communication and support to women at facilities and on WhatsApp, distribution of behavior change communication materials, and mobile phone and mass media messaging. We used logistic regression models adjusted for clustering to measure intervention impact in a cohort of women (*n* = 1200) at 10 intervention and 10 comparison facilities interviewed during their third trimester and at 6 and 24 weeks postpartum.

**Results:**

The intervention significantly increased the percentage of infants who were exclusively breastfed at 6 weeks (83% intervention; 76% comparison; *P* = 0.02) and 24 weeks (66% intervention; 52% comparison; *P* < 0.001), but had no impact on early initiation of breastfeeding (35% intervention; 33% comparison; *P* = 0.65). Among infants who were exclusively breastfed at 6 weeks, the odds of continued exclusive breastfeeding at 24 weeks were higher in the intervention arm than in the comparison arm (OR, 1.6; 95% CI: 1.2-2.1). Infants had increased odds of being exclusively breastfed at 6 weeks if their mothers discussed breastfeeding with a private health provider (OR, 2.3; 95% CI: 1.5-3.4), received text or WhatsApp messages about breastfeeding (OR, 1.7; 95% CI: 1.0-2.7), or heard breastfeeding radio spots (OR, 4.2; 95% CI: 1.2-14.7). Infants had increased odds of exclusive breastfeeding at 24 weeks if their mothers participated in a WhatsApp breastfeeding support group (OR, 1.5; 95% CI: 1.0-2.2).

**Conclusions:**

A breastfeeding intervention in private health facilities in Lagos increased exclusive breastfeeding. Implementation of breastfeeding interventions in private facilities could extend the reach of breastfeeding promotion programs in urban Nigeria. This trial was registered at clinicaltrials.gov as NCT04835051.

## Introduction

Optimal breastfeeding practices, including initiation of breastfeeding within 1 hour of birth and exclusive breastfeeding from birth to 6 months, are important for child survival, health, and development in low- and middle-income countries (LMICs) (1, 2). Globally, only 49% of children <24 months of age start breastfeeding within 1 hour of birth and 44% of infants <6 months of age are exclusively breastfed (3). If these practices could be scaled up to at least 90%, it would prevent 823,000 child deaths per year (4) and help reduce the $340 billion estimated annual cost of not following the WHO and UNICEF breastfeeding guidelines (5).

A variety of factors are known to influence breastfeeding practices, including the sociocultural context; information and support from the health system, family, and community; and individual-level determinants (6, 7). At the sociocultural level, social norms related to breastfeeding (e.g., a belief that infants need water) can be a barrier to optimal breastfeeding (8, 9). At the health-system level, health facilities that lack policies to support breastfeeding or where health-care providers lack training on breastfeeding counseling can negatively impact breastfeeding practices by failing to provide adequate breastfeeding support to women (7, 10, 11). At the community level, family and community members are often key in providing breastfeeding support, but their advice or influence sometimes results in infants not receiving colostrum or being given fluids or foods before 6 months (12–14). At the individual level, women who have weak breastfeeding intentions, have low breastfeeding self-efficacy, face breastfeeding challenges, or are unable to access breastfeeding support during the first few weeks after birth are less likely to have optimal breastfeeding practices (6). To address these types of barriers to breastfeeding, effective interventions often have several components and work at multiple levels simultaneously (6, 15–18) by providing breastfeeding counseling and social support to women through facility- or community-based health workers or support groups, by engaging family members and other key influencers to support breastfeeding, and by providing mass media or other behavior change communication (BCC) (19–24).

A functioning health facility–based strategy for delivering breastfeeding counseling and support to women is essential for breastfeeding promotion in LMICs (7, 25). Health providers are important and trusted sources of breastfeeding information and support during pregnancy and the postpartum period (7). They can play an important role in counseling women and supporting their breastfeeding decisions when women are facing breastfeeding challenges, thereby facilitating the continuation of exclusive breastfeeding (26, 27). To assist health facilities and health providers in providing breastfeeding-related services, the WHO and UNICEF launched the Baby-Friendly Hospital Initiative (BFHI), which includes training on breastfeeding for health providers and a set of guidelines for health facilities to ensure that breastfeeding is actively supported (28). Where it has been partially or fully implemented, BFHI has increased exclusive breastfeeding and, in some settings, early initiation of breastfeeding (29–31). However, implementation of BFHI is far from universal. Approximately 31% of health facilities in LMICs implement BFHI (32), and <5% of facilities in Africa are designated as baby friendly (33). Sustained effort is needed to ensure that BFHI is more widely implemented so that health providers have the knowledge, skills, and confidence to support breastfeeding (10–12). To reach women with breastfeeding support at the health facilities they frequent, the WHO recommends that BFHI be implemented in public and private health facilities (34). Ensuring that private health facilities implement BFHI is important in LMICs, such as Nigeria, where 76% of secondary-level facilities nationwide, including many maternity clinics, are private (35).

The Alive & Thrive initiative in Nigeria is working to increase optimal breastfeeding practices through breastfeeding counseling and support from health providers, breastfeeding promotion and support from community leaders, and mass media campaigns. A population-based household survey conducted for Alive & Thrive in Lagos State in 2017 showed that the prevalence of early initiation of breastfeeding was 45% and the prevalence of exclusive breastfeeding among infants <6 months of age was 57% (36). A health provider survey also conducted in 2017 found that 88% of facilities in Lagos were private (37). Only 40% of midwives, nurses, and doctors involved in breastfeeding promotion were trained on infant and young child feeding (IYCF) in the last 2 years, and 57% said they needed additional training on IYCF. They had inadequate knowledge of optimal breastfeeding practices, and only 43% of them reported counseling individual women on IYCF in the last 6 months.

Little is known about the impact of breastfeeding promotion interventions on breastfeeding practices of women accessing care in private health facilities in Nigeria, although the majority of facilities in the southern part of the country are private (35). This study addresses this gap by evaluating Alive & Thrive's breastfeeding promotion intervention in private health facilities in Lagos State. The main aim of the study was to measure the impacts of the intervention on early initiation of breastfeeding and exclusive breastfeeding at 6 and 24 weeks. The secondary aims were to measure intervention effects on mothers’ breastfeeding knowledge and intentions; examine the association between their exclusive breastfeeding intentions and practices; and test the association between intervention exposures and outcomes.

## Methods

### Study overview

This study was conducted within the same geographic area as an ongoing, cluster-randomized impact evaluation of Alive & Thrive's overall IYCF program in Nigeria (registered at clinicaltrials.gov as NCT02975063). The research described here is a quasi-experimental longitudinal cohort study of women interviewed in their third trimester of pregnancy and at 6 and 24 weeks postpartum to measure breastfeeding intentions and practices (registered at clinicaltrials.gov as NCT04835051). The study was conducted in 20 private health facilities (10 intervention and 10 comparison facilities) in Lagos State, Nigeria. It followed the principles for implementation science in nutrition described by Tumilowicz et al. (38), including initiation and scoping, planning and design, implementation, and discussions of scale-up by the state Ministry of Health.

### Intervention

**FIGURE 1:**
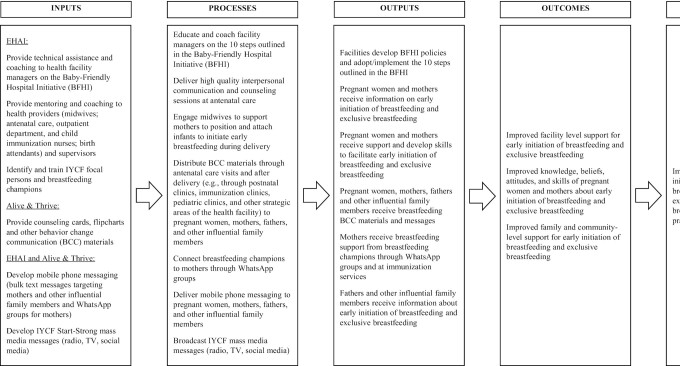
Theory of change for the Alive & Thrive private provider breastfeeding promotion study in Lagos, Nigeria. Abbreviations: BCC, behavior change communication; BFHI, Baby-Friendly Hospital Initiative; EHAI, Equitable Health Access Initiative; IYCF, infant and young child feeding.

The breastfeeding promotion intervention was implemented by Equitable Health Access Initiative in collaboration with Alive & Thrive from May 2019 to April 2020. **Figure 1** shows the project's theory of change, including intervention inputs, processes, outputs, and expected outcomes and impacts. The intervention was designed to strengthen the capacity of private health-care providers to offer high-quality breastfeeding counseling. The intervention consisted of several components: 6 hours of initial and 2 hours of quarterly refresher training for facility managers and staff on implementation of BFHI and breastfeeding counseling skills; provision of interpersonal communication and counseling in person and on WhatsApp by health facility staff; distribution of BCC materials; delivery of mobile phone messages; and broadcasting of mass media messaging. More than 150 facility managers and health-care providers in the antenatal, delivery, immunization, and pediatric outpatient departments at the intervention facilities were trained on BFHI and breastfeeding counseling and support techniques. Facility managers received coaching to develop policies and procedures for implementing the 10 steps of BFHI. Women in the intervention group received in-person breastfeeding counseling and support at the facilities during antenatal, postnatal, and well- and sick-child visits. BCC materials at the facilities included posters and counseling cards, which were part of a state-wide mass media campaign and were provided to health-care providers in the intervention facilities to reinforce IYCF messages. Women received breastfeeding messages on foldable, pocket-sized cards provided to them at the facilities. Women were also invited to participate in WhatsApp breastfeeding support groups, which were organized and managed by the staff member in each intervention health facility who was designated as the breastfeeding champion. The WhatsApp support groups provided women with an opportunity to meet as a group, receive information and support related to breastfeeding, and get answers to their breastfeeding questions. The women and their influential family members (male partners and the women's mothers or mothers-in-law) received text messages. Text messages by phone or WhatsApp were sent in bulk and were 1-way, but WhatsApp support groups offered 2-way communication through the texting function. Women and influential family members gave informed consent to receive the text messages. Mass media television spots on breastfeeding were played on LED screens at the intervention facilities.

In addition to the specific intervention components delivered in intervention facilities during this study, Alive & Thrive's overall IYCF program was ongoing in Lagos State. Alive & Thrive's BCC strategy was based on behavioral science, systems strengthening, and social marketing (39). It included strategic use of data, interpersonal communication, social mobilization, mass media, and policy advocacy (40). Alive & Thrive did not provide support for breastfeeding promotion activities in comparison facilities during this study. Facilities in the comparison areas continued with their usual breastfeeding activities, such as individual breastfeeding counseling offered during provider visits, if at all, with no implementation of BFHI. Alive & Thrive's IYCF program included television and radio spots that were publicly broadcast throughout the state. In the areas where the intervention facilities were located, Alive & Thrive also supported IYCF community mobilization activities, which included sensitization and training of traditional and religious leaders on IYCF to improve awareness of optimal IYCF practices in their communities. In the intervention areas, selected doctors, nurses, midwives, community health extension workers, and traditional birth attendants in public and private health facilities were trained following comprehensive IYCF counseling training manuals to provide counseling to pregnant and breastfeeding mothers. The trained providers were expected to cascade the training to other health providers within their facilities.

### Outcomes

Data were collected during the third trimester of pregnancy and at 6 and 24 weeks postpartum. The main outcomes of this study were early initiation of breastfeeding and exclusive breastfeeding at 6 and 24 weeks, measured using the WHO IYCF questionnaire (41). Secondary outcomes included breastfeeding intentions, collected during the third-trimester survey, and breastfeeding knowledge, collected during the third-trimester, 6-week, and 24-week surveys. Breastfeeding intentions were measured using the breastfeeding duration component of the Infant Feeding Intentions scale (42). Breastfeeding knowledge questions were adapted from Alive & Thrive impact evaluations in Nigeria and other countries, and they used an open-response format (19–21, 36).

### Sampling and Participant Eligibility

The study was conducted in 20 private health facilities purposefully selected from the intervention and comparison areas that had been assigned as part of the overall Alive & Thrive Nigeria impact evaluation. For this study, we chose 10 facilities in the intervention area and 10 in the comparison area that provided maternity and pediatric services, such as antenatal care, postnatal care, and immunizations; were registered with the Association of General and Private Medical Practitioners of Nigeria and the Health Facility Monitoring and Accreditation Agency; had a monthly average of ≥40 antenatal clients and ≥20 deliveries over 3 months; and agreed to participate in the research. The selected facilities provided the research team with the current number of pregnant women in their third trimester. In the 3 lowest-volume facilities (i.e., those with <35 eligible clients) in each study arm, we attempted to enroll all pregnant women in their third trimester. For all other facilities, the number of women sampled was proportional to the facility size within each study arm. All eligible women attending antenatal care on the days of data collection were invited to participate until the target enrollment at each facility was achieved.

Women who were attending antenatal care at the study facilities were approached by interviewers in the waiting room and invited to a private area for recruitment into the study. Women were eligible to enroll if they were ≥18 years old, in their third trimester of pregnancy, and current clients of a private health facility selected for the study. At 6 and 24 weeks postpartum, the women continued to be eligible if their infant was alive and neither the mother nor infant had a health condition that caused breastfeeding to be contraindicated. Women remained in the study regardless of where they sought postnatal care or well-child services.

### Sample size

We calculated the sample size for our 2 main outcome variables—early breastfeeding initiation and exclusive breastfeeding—using prevalence estimates from Alive & Thrive's 2017 population-based survey (36). We adopted the larger required sample size, which was for early breastfeeding initiation. To detect a difference of 8 percentage points (pp; from 45% to 53%) in the proportion of women who initiated breastfeeding within 1 hour of delivery in the intervention and comparison health facilities with 80% power, an α of 0.05, and an intraclass correlation of 0.005, we required 960 women across a total of 20 health facilities, which are considered clusters. We added 25% to the sample for attrition because the study took place in an urban area where we expected participants to be challenging to track. This resulted in a final sample size of 1200 women (600 per study arm).

### Data collection

The evaluation was conducted by RTI International and Datametrics Associates Ltd. Data collectors were not blinded to the evaluation design because Alive & Thrive BCC materials were visible in intervention facilities. The data collection team was divided into groups of 3 to 4 interviewers and 1 supervisor. Interviewers and supervisors were trained for 5 days before the first round of data collection (i.e., during the women's third trimester). They participated in a 3-day refresher training before 6-week postpartum data collection and a 3-day refresher before 24-week postpartum data collection. Each training included a pilot of data collection procedures.

Survey questionnaires were used to obtain data on women's breastfeeding intentions (third trimester) and practices (6 and 24 weeks), their breastfeeding knowledge, their exposure to the intervention, and demographic characteristics. Intervention exposures to interpersonal communication at the facility, mobile phone messaging and support, and mass media were measured at each survey wave. Demographic questions were taken from the Nigeria Demographic and Health Survey (43), and the household hunger scale was used to measure food security (44). Data collection tools were translated into Yoruba by 2 translators, who translated the tools separately, reviewed each other's translations, identified differences, and agreed on the best translation. The questionnaire was available in English and Yoruba.

Interviewers administered the questionnaires and entered the responses into electronic tablets using Open Data Kit. Supervisors spot-checked 10% of the questionnaires and reviewed all completed questionnaires before uploading them to the server. The majority of the interviews were conducted face to face. Interviewers conducted approximately one-quarter of the 24-week interviews by phone because travel restrictions related to the coronavirus disease 2019 pandemic were put in place in Lagos in mid-March 2020. Interviewers already had the participants’ phone numbers, which they had been using to make appointments for the 6-week and 24-week interviews.

Ethical approval for the research was obtained from the RTI International institutional review board and the Lagos State University Teaching Hospital Health Research and Ethics Committee, and permission was obtained for phone-based data collection prior to transitioning during the pandemic. The consent form was read aloud to participants, and they provided written consent at enrollment.

### Data analysis

All analyses were conducted in Stata MP Version 16.0 using survey commands to account for clustering effects within facilities. At each round of data collection, data were analyzed cross-sectionally using regression models to determine the intervention impact on breastfeeding knowledge, intentions, and practices and the association of specific intervention exposures with breastfeeding outcomes. Exposure variables for 6 and 24 weeks were constructed so that they were cumulative from the start of the intervention. Tests for differences were not conducted for variables with cell sizes less than 5, except for third-trimester analyses of maternal education and household hunger, which were performed using clustered χ² tests. Longitudinal models were fit to examine the relationship between breastfeeding intentions, measured during the third trimester, and exclusive breastfeeding practices at 6 and 24 weeks.

To determine whether adjustments to regression models were needed to correct for unintentional bias that may have occurred from the nonrandomized design, the demographic characteristics of participants measured during the third trimester were used to calculate inverse probability weights. We found that applying inverse probability weights was not necessary to balance the study arms for key outcome variables; therefore, we conducted the analysis without adjustment.

Women who missed their 6-week postpartum interview were allowed to rejoin the sample at 24 weeks. Characteristics of women interviewed at all 3 time points were compared with women interviewed at 1 or 2 time points. There were no differences between those with and without missing surveys at the *P* < 0.05 level with respect to key outcomes and demographic variables.

## Results

### Sample and participant characteristics

**FIGURE 2:**
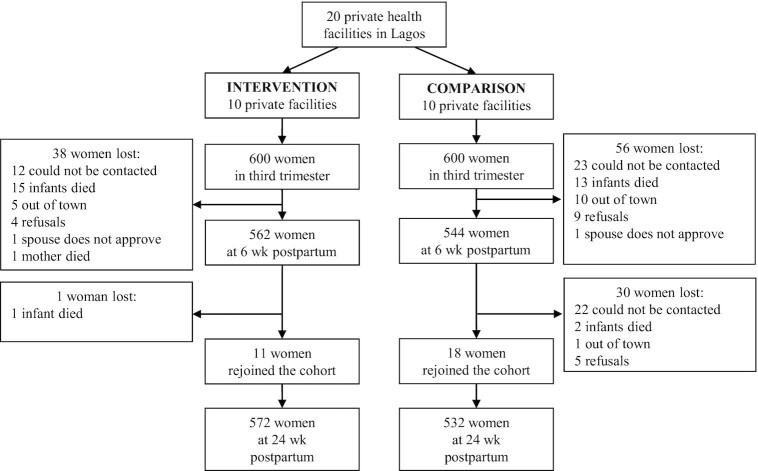
Study flow diagram for the Alive & Thrive private provider breastfeeding promotion study in Lagos, Nigeria.

Of the 600 women enrolled in each study arm during their third trimester of pregnancy, 562 remained in the intervention arm and 544 remained in the comparison arm at 6 weeks (**Figure 2**). At 24 weeks, 572 were included in the intervention arm and 532 in the comparison arm. The percentages of women enrolled by facility within each study arm are shown in **Supplemental Table 1**. At enrollment during the third trimester, the women's characteristics did not differ by study arm (**Table 1**). On average, women in the sample were 30 years old and had 2.2 children. Nearly all women in the sample were married, and their households had little to no household hunger. Most of the women were small traders/self-employed or salaried nongovernment employees, and the majority had partially or fully completed their postsecondary education. Approximately 90% of the women in both study arms delivered their babies in the private facilities. The percentages of women whose baby was delivered by caesarean section did not differ by study arm (24% intervention; 25% comparison; *P* = 0.92).

**TABLE 1:** Characteristics at enrollment during third trimester of women participating in the Alive & Thrive private provider study in Lagos, Nigeria

	Intervention (*n* = 600)	Comparison (*n* = 600)	*P* value
Marital status, % (*n*)	—	—	0.69
Married	99 (594)	99 (592)	
Single	1 (6)	1 (8)	
Primary occupation, % (*n*)	—	—	0.37
Salary government employee	6 (37)	5 (32)	
Salary nongovernment employee	22 (132)	28 (169)	
Small trader/self-employment	56 (333)	48 (290)	
Housewife	11 (68)	10 (59)	
Jobless	2 (10)	4 (24)	
Pupil/student	2 (13)	3 (16)	
Other	1 (7)	2 (10)	
Level of education, % (*n*)	—	—	0.89
Completed primary or less	2 (9)	2 (12)	
Partial or completed secondary	36 (217)	31 (183)	
Partial or completed postsecondary	59 (351)	56 (336)	
Masters or Doctorate	3 (19)	6 (34)	
Other	1 (3)	6 (35)	
Participant ethnicity, % (*n*)	—	—	0.48
Yoruba	34 (202)	39 (234)	
Igbo	44 (262)	42 (252)	
Other	23 (136)	19 (114)	
Household hunger, % (*n*)	—	—	0.96
Little to no household hunger	99 (595)	99 (591)	
Moderate household hunger	1 (5)	1 (8)	
Severe household hunger	0 (0)	0 (1)	
Age, years, mean (SE)	30.3 (0.4)	30.3 (0.6)	0.90
Total number of rooms in household, mean (SE)	4.5 (0.2)	5.0 (0.3)	0.19
Household assets, maximum 34 items, mean (SE)	15.4 (0.5)	16.5 (0.7)	0.21

### Impact of the intervention on breastfeeding practices

**FIGURE 3:**
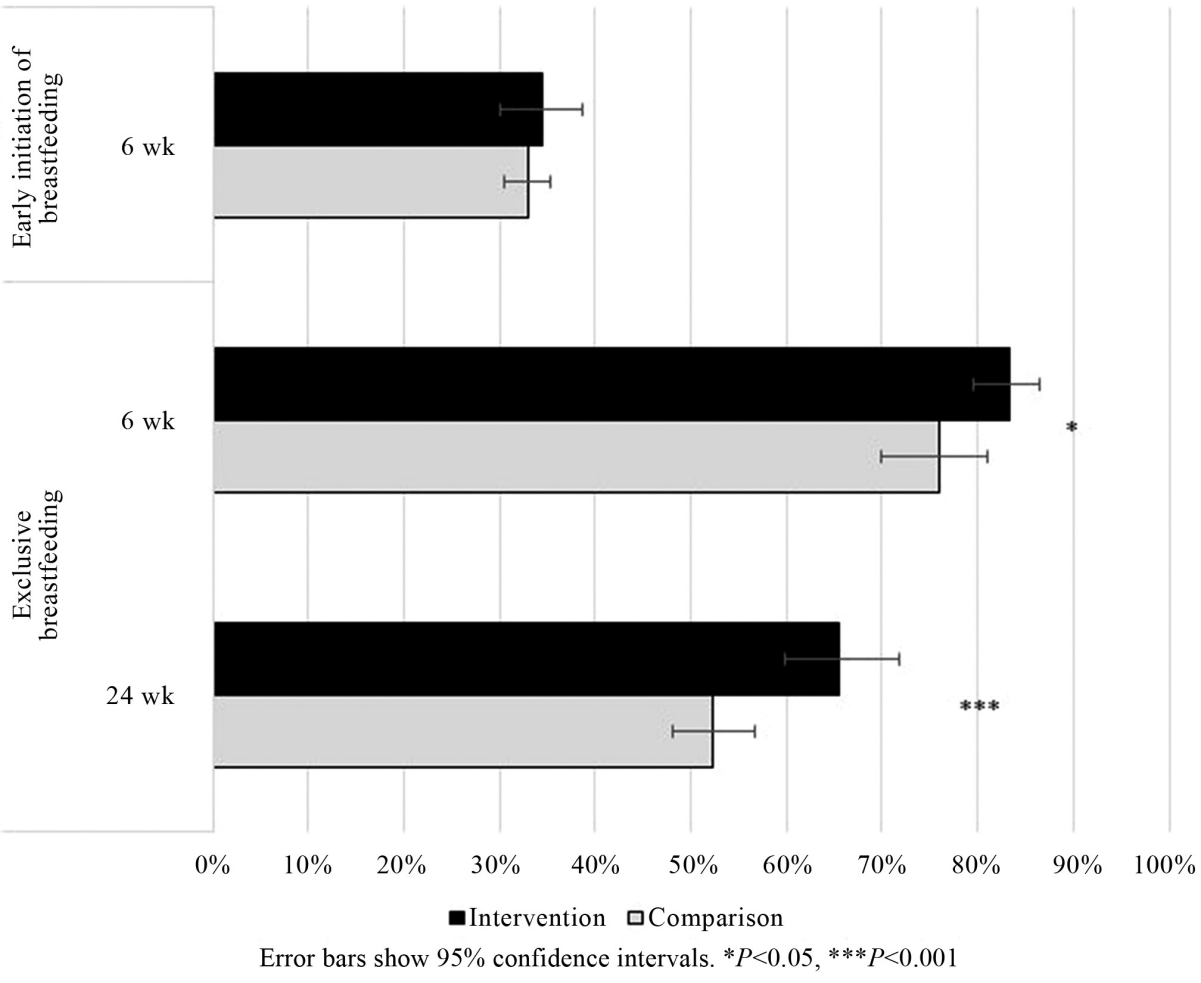
Impact of the Alive & Thrive breastfeeding intervention in private health facilities on early initiation of breastfeeding and exclusive breastfeeding.

Approximately one-third of the women reported that they initiated breastfeeding within 1 hour of delivery, and there was no statistically significant difference by study arm (35% intervention; 33% comparison; +2 pp; *P* = 0.65; **Figure 3**). Significantly higher percentages of women in the intervention than the comparison arm exclusively breastfed their infants at 6 weeks (83% intervention; 76% comparison; +7 pp; *P* = 0.02) and at 24 weeks (66% intervention; 52% comparison; +14 pp; *P* < 0.001). Infants in the intervention arm who were exclusively breastfed at 6 weeks had increased odds of continued exclusive breastfeeding at 24 weeks compared with infants in the comparison arm (OR, 1.6; 95% CI: 1.2–2.1).

### Impact of the intervention on breastfeeding knowledge and intentions

Knowledge of recommended breastfeeding practices was generally high among women in both study arms. During the third trimester, a higher percentage of women in the intervention than comparison arm knew that the baby should be breastfed within 1 hour of delivery (+6 pp; *P* = 0.004) and that mothers should give colostrum to the baby soon after birth (+9 pp; *P* = 0.004; **Table 2**). Knowledge of these topics did not differ by study arm at 6 or 24 weeks. Most women knew the correct definition of exclusive breastfeeding, and the percentage did not differ by study arm at any point during the study. More women in the intervention arm knew that liquids other than breast milk should not be given to infants before 6 months, both when asked during the third trimester (+12 pp; *P* = 0.007) and at 6 weeks (+21 pp; *P* = 0.04). Similarly, more women in the intervention arm knew that food should not be given to infants before 6 months, both when asked during the third trimester (+17 pp; *P* = 0.01) and at 6 weeks (+19 pp; *P* = 0.06). We found no differences by study arm in these indicators of breastfeeding knowledge at 24 weeks.

**TABLE 2:** Breastfeeding knowledge and intentions of women participating in the Alive & Thrive private provider study in Lagos, Nigeria

	Third trimester					6 weeks					24 weeks				
	Intervention (*n* = 600)		Comparison (*n* = 600)		pp difference	Intervention (*n* = 562)		Comparison (*n* = 544)		pp difference	Intervention (*n* = 572)		Comparison (*n* = 532)		pp difference
	%	*n*	%	*n*		%	*n*	%	*n*		%	*n*	%	*n*	
Breastfeeding knowledge															
Infant should start breastfeeding within 1 hour of birth	96	575	90	537	6^1^	94	530	95	518	−1	90	512	92	491	−2
Colostrum should be given to the infant soon after birth	91	543	82	492	9^1^	99	558	99	541	0	100	572	100	530	0
Exclusive breastfeeding means giving infant only breast milk up to 6 months	95	571	94	564	1	99	557	100	542	−1	99	569	100	531	−1
Infants should start receiving liquids other than breast milk from 6 months	50	297	38	227	12^1^	40	238	19	115	21^2^	39	234	23	140	16
Infants should start receiving food in addition to breast milk from 6 months	75	448	58	346	17^2^	64	382	45	270	19^3^	58	349	48	287	10
Breastfeeding intentions^4^															
Intends to exclusively breastfeed at 6 weeks	92	551	92	553	0	—	—	—	—	—	—	—	—	—	—
Intends to exclusively breastfeed at 24 weeks	78	466	64	384	14^5^	—	—	—	—	—	—	—	—	—	—

Abbreviation: pp, percentage point.

1 *P* < 0.10.

2 *P* < 0.05.

3 *P* < 0.01.

4 *P* < 0.001.

5 Breastfeeding intentions were measured during the third trimester.

The intention to exclusively breastfeed at 6 weeks postpartum did not differ by study arm, whereas the intention to exclusively breastfeed at 24 weeks postpartum was higher in the intervention arm (+14 pp; *P* < 0.001).

### Relationship between breastfeeding intentions and practices

The intention to exclusively breastfeed at 6 weeks was associated with increased odds of exclusive breastfeeding at that time (OR, 3.1; 95% CI: 1.7–5.7). Similarly, the intention to exclusively breastfeed at 24 weeks was associated with increased odds of exclusive breastfeeding at that time (OR, 2.8; 95% CI: 2.1–3.8).

### Exposure to the intervention and association of exposures with outcomes

Using data on exposure at each time point, no statistically significant differences by study arm were observed in the percentages of women who received breastfeeding counseling from a health provider at a private facility (**Supplemental Table 2**). Significantly more women in the intervention than comparison arm received take-home BCC materials about breastfeeding during the third trimester; saw Alive & Thrive television messages in the third trimester and at 6 and 24 weeks; and heard Alive & Thrive radio messages at 6 weeks.

Using data on cumulative intervention exposure in the intervention arm, more women were exposed to counseling or advice from a provider at a private facility than from any other intervention component, with 93% of the sample reporting counseling by 24 weeks (**Table 3**). Approximately one-quarter of the women received take-home BCC materials during pregnancy, and few additional women received them thereafter. About one-quarter of the women received text or WhatsApp messages during pregnancy, increasing to nearly half of the women at 24 weeks. Women reported that very few of their husbands or mothers/mothers-in-law received text messages about breastfeeding (data not shown). Women's participation in WhatsApp groups was low during pregnancy, but the percentage more than doubled by 24 weeks. Approximately one-quarter of the women reported seeing Alive & Thrive television spots during pregnancy, increasing to two-thirds of the women at 24 weeks. Women's exposure to Alive & Thrive radio spots was the lowest of all intervention components but increased over time.

**TABLE 3:** Exposure of women in the intervention arm to Alive & Thrive intervention components provided through private health facilities, mobile phones, and mass media and association of exposures to breastfeeding outcomes

	Cumulative Intervention Exposure^1^						Association of Exposures to Outcomes					
	Third trimester (*n* = 600)		6 weeks (*n* = 562)		24 weeks (*n* = 572)		Early initiation of breastfeeding		Exclusive breastfeeding 6 weeks		Exclusive breastfeeding 24 weeks	
	%	*n*	%	*n*	%	*n*	OR	95% CI	OR	95% CI	OR	95% CI
At private facility												
Health-care provider at a private facility spoke about breastfeeding	74	444	88	492	93	531	2.3^2^	1.1–4.8	2.3^3^	1.5–3.4	1.1	0.5–2.4
Received take-home BCC materials about breastfeeding	24	141	26	145	30	171	1.0	0.7–1.4	0.8	0.5–1.4	0.9	0.6–1.4
Through mobile phone												
Received text or WhatsApp messages about breastfeeding	23	136	42	233	48	237	1.4^2^	1.0–1.9	1.7^2^	1.0–2.7	1.3	0.8–2.0
Participated in a WhatsApp breastfeeding group	18	108	35	196	42	239	1.1	0.8–1.6	1.3	0.8–2.1	1.5^2^	1.0–2.2
Through mass media												
Saw Alive & Thrive spots on television in last 30 days	26	153	47	265	67	382	0.9	0.6–1.4	1.7	0.9–3.3	1.1	0.7–1.9
Heard Alive & Thrive spots on radio in last 30 days	8	45	14	76	21	117	1.2	0.6–2.3	4.2^2^	1.2–14.7	1.3	0.7–2.5

Abbreviation: BCC, behavior change communication.

1 Intervention exposure at 6 weeks and 24 weeks was calculated cumulatively from the start of the intervention.

2 *P* < 0.05.

3 *P* < 0.001.

Infants had increased odds of starting breastfeeding within 1 hour of birth if a health provider at the private facility spoke with their mother about breastfeeding (OR, 2.3; 95% CI: 1.1–4.8) or their mother received text or WhatsApp messages about breastfeeding (OR, 1.4; 95% CI: 1.0–1.9). Infants had increased odds of exclusive breastfeeding at 6 weeks if a health provider spoke with their mother about breastfeeding (OR, 2.3; 95% CI: 1.5–3.4), their mother received text or WhatsApp messages about breastfeeding (OR, 1.7; 95% CI: 1.0–2.7), or they heard Alive & Thrive radio spots (OR, 4.2; 95% CI: 1.2–14.7). Infants had increased odds of exclusive breastfeeding at 24 weeks if their mother participated in a WhatsApp breastfeeding group (OR, 1.5; 95% CI: 1.0–2.2).

## Discussion

This study showed that a breastfeeding promotion intervention implemented in private health facilities in Lagos, Nigeria, increased exclusive breastfeeding and women's knowledge of and intentions related to it. The intervention also increased women's knowledge of early initiation of breastfeeding, but did not change this practice. To our knowledge, this is the first study to show an effect of a breastfeeding promotion intervention in private health facilities in an LMIC. It indicates that private health facilities can successfully implement activities to actively promote breastfeeding among their clients.

While interventions in health facilities or interventions that included a health facility component in LMICs consistently have had an impact on exclusive breastfeeding, results on early initiation have been more mixed. For example, Alive & Thrive's intervention in Bangladesh, Burkina Faso, and Ethiopia, which included breastfeeding counseling by public health providers or community health workers, had impacts on both exclusive breastfeeding and early initiation of breastfeeding (19, 21, 45), whereas in Vietnam it had an impact on exclusive breastfeeding but not on early initiation of breastfeeding (19). Studies of BFHI implementation in public health facilities have shown effects on exclusive breastfeeding, but many have not measured early initiation using the standard WHO indicator (31). Among BFHI studies in LMICs that measured both breastfeeding outcomes, a study in Brazil found increases in early initiation of breastfeeding and exclusive breastfeeding (30), while a study in the Democratic Republic of Congo found an increase in exclusive breastfeeding but not in early initiation of breastfeeding (29). The impact of some breastfeeding interventions, including the intervention evaluated in this study, on exclusive breastfeeding but not on early initiation of breastfeeding may be related to the differences in the determinants of these 2 outcomes. Early initiation of breastfeeding is a discrete behavior that occurs shortly after delivery and is influenced by the place of delivery, type of delivery, delivery complications, who assists with the delivery, size of the baby, and socioeconomic status (46, 47). In Nigeria, beliefs about the need to clean the baby and mother before starting to breastfeed may also delay breastfeeding initiation (14). Exclusive breastfeeding takes concerted effort over a period of time and is influenced by mother and infant attributes, mother-infant interactions, family and community support, health system support and services, workplace and employment factors, and the sociocultural context (6). More research is needed to understand how determinants of early initiation of breastfeeding can be influenced to consistently modify the practice through BFHI or other breastfeeding promotion programs in public or private health facilities.

Breastfeeding intentions are usually established by the third trimester of pregnancy and are associated with breastfeeding practices (48–50), as corroborated by this study. We found that women's intentions to exclusively breastfeed at 24 weeks postpartum differed by study arm, and more women in the intervention arm exclusively breastfed their infants at that time. This shows that the support offered through the intervention was important for reinforcing intentions and ensuring that women followed through.

The intervention in this study included the implementation of BFHI in private health facilities but also went beyond the 10 steps to successful breastfeeding by including interpersonal communication between providers and clients during postnatal and child health visits and incorporating a mobile phone component to support breastfeeding groups and to allow direct communication between providers and clients outside the facility setting. Our findings align with results from reviews showing that BFHI implementation and interventions in LMICs that include face-to-face communication and/or in-person or phone support delivered by lay people, health professionals, or a combination improved breastfeeding practices (12, 31, 51). Multi-component breastfeeding promotion interventions have proven effective in LMICs because they influence different factors that drive breastfeeding practices, including knowledge, intentions, beliefs, social norms, and self-efficacy (17, 19–22, 24, 45, 49).

Exposure and intensity are important components of breastfeeding interventions (19, 22). In this study, in-person interpersonal communication by private health providers was the most common exposure throughout the intervention and was associated with early initiation of breastfeeding and exclusive breastfeeding at 6 weeks. The link between interpersonal communication and breastfeeding practices aligns with findings from Alive & Thrive evaluations in Burkina Faso and Vietnam (22, 45). Exposure to mobile phone and mass media intervention components was low during pregnancy and ramped up throughout the intervention, but remained below 50% coverage for most of these types of exposures at 24 weeks. Breastfeeding text messages were associated with early initiation of breastfeeding and exclusive breastfeeding at 6 weeks, while WhatsApp support group participation was associated with exclusive breastfeeding at 24 weeks. The association of text and mobile phone support with breastfeeding practices is consistent with the results of the only published study in an LMIC where health providers used phones to support breastfeeding (52).

This study's strengths included the cohort design and lower-than-expected attrition, which provided more statistical power to detect intervention effects. This study also had some limitations. We could not randomly assign facilities to study arms, because this research took place within the context of an ongoing impact evaluation of Alive & Thrive's overall program in Lagos State, which included random assignment of geographical areas. Therefore, facilities for this study were selected within the intervention and comparison areas of the overall impact evaluation. To account for the lack of random assignment, we assessed whether an inverse probability weighting adjustment based on women's demographic characteristics during pregnancy was needed. We found that the adjustment was unnecessary to establish balance for key outcome variables. It is possible that our findings were affected by the study being conducted in Lagos, where Alive & Thrive's overall intervention was ongoing. However, in the private provider study, we carefully measured women's exposure to all Alive & Thrive intervention components and found that their participation in Alive & Thrive's overall interpersonal communication and community mobilization activities was low. One other limitation of this study was the lack of random selection of facilities, because we required medium-to-large or high-volume facilities to achieve our sample size. Therefore, the findings may not be generalizable to small or low-volume private facilities.

In conclusion, this study demonstrates that training health-care providers in private health facilities on BFHI, providing breastfeeding counseling, and offering mobile phone support for breastfeeding increases exclusive breastfeeding among their clients. The size of the intervention effect on exclusive breastfeeding was large enough to make the intervention worth expanding to other private facilities in Lagos and in other cities in Nigeria. To facilitate this process, implementers should provide a detailed description of implementation strategies in the intervention package to the Lagos State and Federal Ministries of Health. Further analysis of the monitoring and process data collected during the study is needed to adapt the intervention to ensure better uptake of early initiation of breastfeeding and more involvement of influential family members. Implementation research in smaller private health facilities should also be conducted to learn how to modify the intervention for such settings. The intervention could be tested in other LMICs where private health providers and maternity hospitals are common to determine whether it is equally effective and whether other modifications are necessary. Expanding the repertoire of breastfeeding interventions in private health facilities in LMICs will help to achieve global breastfeeding coverage targets (53).

## Supplementary Material

nxab450_Supplemental_File

## Notes

This research was funded by the Alive & Thrive initiative, managed by FHI Solutions, and currently funded by the Bill and Melinda Gates Foundation (OPP 1135932, INV-029432), Irish Aid, the Tanoto Foundation, UNICEF, and the World Bank.

Author disclosures: UR-O and SB are employed by Alive & Thrive. OA is employed by the Equitable Health Access Initiative.

Alive & Thrive and Equitable Health Access Initiative were involved in the design, implementation, and monitoring of the intervention, but not in data collection or analysis for this study. All other authors report no conflicts of interest.

Supplemental Tables 1 and 2 are available from the “Supplementary data” link in the online posting of the article and from the same link in the online table of contents at https://academic.oup.com/jn/.

Abbreviations used: BCC, behavior change communication; BFHI, Baby-Friendly Hospital Initiative; IYCF, infant and young child feeding; LMICs, low- and middle-income countries; pp, percentage point.
