# Supplementary material for: Breastfeeding Interpersonal Communication, Mobile Phone Support, and Mass Media Messaging Increase Exclusive Breastfeeding at 6 and 24 Weeks Among Clients of Private Health Facilities in Lagos, Nigeria
Source: J Nutr. 2022 Jan 7;152(5):1316–26. doi: 10.1093/jn/nxab450 (PMC9071272; doi:10.1093/jn/nxab450)
Supplement: nxab450_Supplemental_File [file nxab450_supplemental_file.zip › Supplemental Table 1_revised.docx]

Supplemental Table 1: Alive & Thrive Lagos private provider study sample by health facility at enrollment

| Facility Name (Local Government Area^1^) | % | N |
| --- | --- | --- |
| *Intervention Facilities* |  | (N=600) |
| Mobonike Hospital (Agege) | 22.7 | 136 |
| Promise Medical Centre (Agege) | 14.3 | 86 |
| Eko Hospital (Ikeja) | 1.5 | 9 |
| Isalu Hospital (Ikeja) | 3.8 | 23 |
| Albina Mejeel Specialist Clinic (Ojo) | 8.2 | 49 |
| Ilogbo Central Hospital (Ojo) | 13.5 | 81 |
| R-Jolard (Somolu) | 25.5 | 153 |
| RCCG Maternity Home Okesuna (Somolu) | 6.5 | 39 |
| Uwemedimo Hospital (Surulere) | 2.3 | 14 |
| RCCG Ebute Metta Maternity Home (Mainland) | 1.7 | 10 |
| *Comparison Facilities* |  | (N=600) |
| Faith City Hospital (Oshodi) | 2.0 | 12 |
| Edmac Medical Center (Oshodi) | 5.3 | 32 |
| Crystal Specialist Hospital (Alimosho) | 34.3 | 206 |
| El-Dunamis Medical Centre (Alimosho) | 11.3 | 68 |
| Matador Medical Service (Ifako Ijaye) | 2.2 | 13 |
| Ayodele Medical Centre (Ifako Ijaye) | 8.5 | 51 |
| Modupe Oluwa Maternity Home (Mushin) | 9.2 | 55 |
| Regina Mundi Catholic Hospital (Mushin) | 11.2 | 67 |
| Fuja Medical Center (Lagos Island) | 2.3 | 14 |
| Redeemed Maternity (Amuwo) | 13.7 | 82 |

^1^Local government area, the largest administrative subunit within a state in Nigeria.
