# Supplementary material for: Breastfeeding Interpersonal Communication, Mobile Phone Support, and Mass Media Messaging Increase Exclusive Breastfeeding at 6 and 24 Weeks Among Clients of Private Health Facilities in Lagos, Nigeria
Source: J Nutr. 2022 Jan 7;152(5):1316–26. doi: 10.1093/jn/nxab450 (PMC9071272; doi:10.1093/jn/nxab450)
Supplement: nxab450_Supplemental_File [file nxab450_supplemental_file.zip › Supplemental Table 2_revised.docx]

Supplemental Table 2: Women’s exposure to interpersonal communication and Alive & Thrive mass media in the intervention and comparison arms of the Alive & Thrive Lagos private provider study^1^

|  | Third trimester | | | | | 6 weeks | | | | | 24 weeks | | | |  |
| --- | --- | --- | --- | --- | --- | --- | --- | --- | --- | --- | --- | --- | --- | --- | --- |
|  | Intervention  (N=600) | | Comparison  (N=600) | | Intervention  (N=562) | | | Comparison  (N=544) | | Intervention  (N=572) | | | Comparison  (N=532) | |  |
|  | % | N | % | N | % | | N | % | N | % | | N | % | N |  |
| **INTERPERSONAL COMMUNICATION EXPOSURE AT A PRIVATE FACILITY** |  |  |  |  |  | |  |  |  |  | |  |  |  |  |
| Health care provider at a private facility spoke about breastfeeding | 74 | 444 | 62 | 369 | 51 | | 306 | 38 | 229 | 59 | | 354 | 51 | 304 |  |
| Received take-home BCC materials about breastfeeding | 24 | 141 | 6*** | 36 | 4 | | 21 | 3 | 19 | 7 | | 40 | 3 | 17 |  |
| **EXPOSURE THROUGH MASS MEDIA** |  |  |  |  |  | |  |  |  |  | |  |  |  |  |
| Saw Alive & Thrive ads on television in last 30 days | 26 | 153 | 12** | 72 | 28 | | 166 | 12** | 72 | 44 | | 263 | 27** | 159 | |
| Heard Alive & Thrive ads on radio in last 30 days | 8 | 45 | 4 | 26 | 7 | | 40 | 2** | 14 | 9 | | 55 | 7 | 39 | |

^1^Exposures were measured at each time point and are not cumulative. **P*<0.05; ***P*<0.01; ****P*<0.001; BCC, behavior change communication
